# Three-dimensional exoscope-assisted combined petrosal approach for petroclival meningiomas: technical nuances from early experience

**DOI:** 10.3389/fonc.2026.1851935

**Published:** 2026-08-19

**Authors:** Alessandro Pesaresi, Ryokichi Yagi, Leonardo Bradaschia, Nicola Pio Fochi, Enrico Lo Bue, Alice Antico, Silvia Sgambetterra, Camilla Origlia, Diego Garbossa, Federica Penner, Francesco Zenga

**Affiliations:** 1 Skull Base and Pituitary Surgery Unit, “Città della Salute e della Scienza” University Hospital, Turin, Italy; 2 Neurosurgery Unit, “Città della Salute e della Scienza” University Hospital, Turin, Italy; 3 Department of Neuroscience “Rita Levi Montalcini”, University of Turin, Turin, Italy; 4 Department of Neurosurgery and Neuroendovascular Therapy, Osaka Medical and Pharmaceutical University, Osaka, Japan

**Keywords:** combined petrosal approach, exoscope, meningioma, petroclival meningioma, petrosectomy, skull base

## Abstract

**Introduction:**

Petroclival meningiomas (PCMs) are among the most challenging skull base tumors due to their deep location and proximity to critical neurovascular structures. The combined petrosal approach (CPA) provides wide exposure of the petroclival region, although conventional microscopic visualization may be ergonomically demanding in narrow operative corridors. The introduction of three-dimensional (3D) exoscopes offers enhanced magnification, improved ergonomics, and shared visualization. This study evaluates the feasibility and preliminary outcomes of CPA for PCM resection performed using a 4K 3D exoscope during its first year of clinical application.

**Methods:**

Three consecutive patients underwent CPA for PCM resection using the ORBEYE™ 4K 3D exoscope between December 2024 and December 2025. Clinical data included demographics, pre- and postoperative neurological deficits, complications, operative time, extent of resection, and the 3-month outcomes. Gross total resection (GTR) was defined as the absence of residual enhancing tumor on postoperative MRI, whereas subtotal resection (STR) indicates intentional residual tumor left to minimize the risk of neurological injury.

**Results:**

The mean age at surgery was 50.7 years. All patients presented with preoperative cranial nerve (CN) deficits. GTR was achieved in two cases, while one patient underwent planned STR followed by radiosurgery. The mean operative time was 9 h and 40 min. One transient postoperative CN VI palsy completely resolved within 3 months, and one patient developed pneumonia that was successfully treated during hospitalization. No intraoperative complications or mortality occurred.

**Conclusions:**

Although the limited sample precludes conclusions with regard to efficacy or superiority, our preliminary experience suggests that the 4K 3D exoscope is a feasible adjunct in CPA for PCM surgery, providing high-quality visualization and favorable early operative outcomes.

## Introduction

Petroclival meningiomas (PCMs) are among the most challenging skull base tumors to manage surgically due to their deep-seated location and proximity to critical neurovascular structures (1). The combined petrosal approach (CPA), which integrates anterior and posterior petrosal techniques, offers extensive exposure of the petroclival region, facilitating maximal tumor resection while aiming to preserve neurological function (2).

Traditional microsurgical methods, while effective, can be limited by ergonomic constraints and suboptimal visualization, especially in deep and narrow operative corridors. The advent of a high-definition (HD) three-dimensional (3D) exoscope has introduced a paradigm shift in neurosurgical visualization, offering enhanced magnification, improved ergonomics, and the potential for better surgical outcomes (3). This paper explores the integration of intraoperative exoscope technology into the CPA for PCM resection, evaluating its impact on surgical efficacy and patient outcomes in a consecutive case series of patients.

## Methods

Data from patients undergoing CPA for PCM using a 3D exoscope over a period of 1 year (between December 2024 and December 2025) at the authors’ institution were collected. All procedures were performed by the same senior surgeon (FZ). Data regarding gender, age at surgery, preoperative neurological deficits, new-onset neurological deficits, postoperative complications, operating room time, extent of resection (EoR), and neurological deficits at 3 months after surgery were collected. Gross total resection (GTR) was defined as the absence of residual enhancing tumor on postoperative contrast-enhanced MRI, near-total resection (NTR) as a resection >90% of the tumor volume, and partial resection (PR) as intentional residual tumor left adherent to critical neurovascular structures with a resection <90% of the preoperative volume. Due to the retrospective design of the study, the requirement for Institutional Review Board (IRB) approval was formally waived by the local Ethics Committee at the City of Health and Science Hospital of Turin. Written informed consent for treatment was obtained from all patients. All data were obtained from medical records collected during routine clinical practice, which were fully anonymized prior to analysis, and handled in accordance with institutional and national regulations and the principles of the Declaration of Helsinki.

### ORBEYE^TM^ 4K 3D exoscope design

The ORBEYE™ 4K 3D exoscope system (Olympus, Tokyo, Japan) is the first to combine ultra-high-definition (4K) clarity with 3D visualization. It is composed of two metal oxide semiconductor cameras, each with a resolution of 3,840 × 2,160 pixels. Each camera has an individual counterbalance arm operated by a traditional dead man’s switch. The camera body itself contains the dead man’s switch and the controls for zoom and focus. The exoscope and the counterbalance arms are mounted on a console. The operating system of the exoscope is Unix-like and is controlled via a graphical user interface integrated within the console. Illumination of the field is provided by a fiber-optic LED source. The system generates its image via a 3D line-by-line mode that can be displayed on compatible 3D monitors using the same resolution as that of the capturing cameras. The image is a full-resolution circularly polarized passive 3D image that requires the observer to wear circularly polarized 3D glasses for proper visualization. Optical and digital zooming can be performed using either a hand or a foot switch located on the exoscope. The magnification ranges from ×1.1 to ×25.8, with a zoom ratio of 1:12 (a sixfold optical zoom and a twofold digital zoom). This exoscope has a focal length of 220–550 mm, and focus can be adjusted via the exoscope headpiece or a foot pedal. The field of view ranges from 7.5 to 171 mm (4).

### Surgical procedure

#### Intraoperative setting

**Figure 1:**
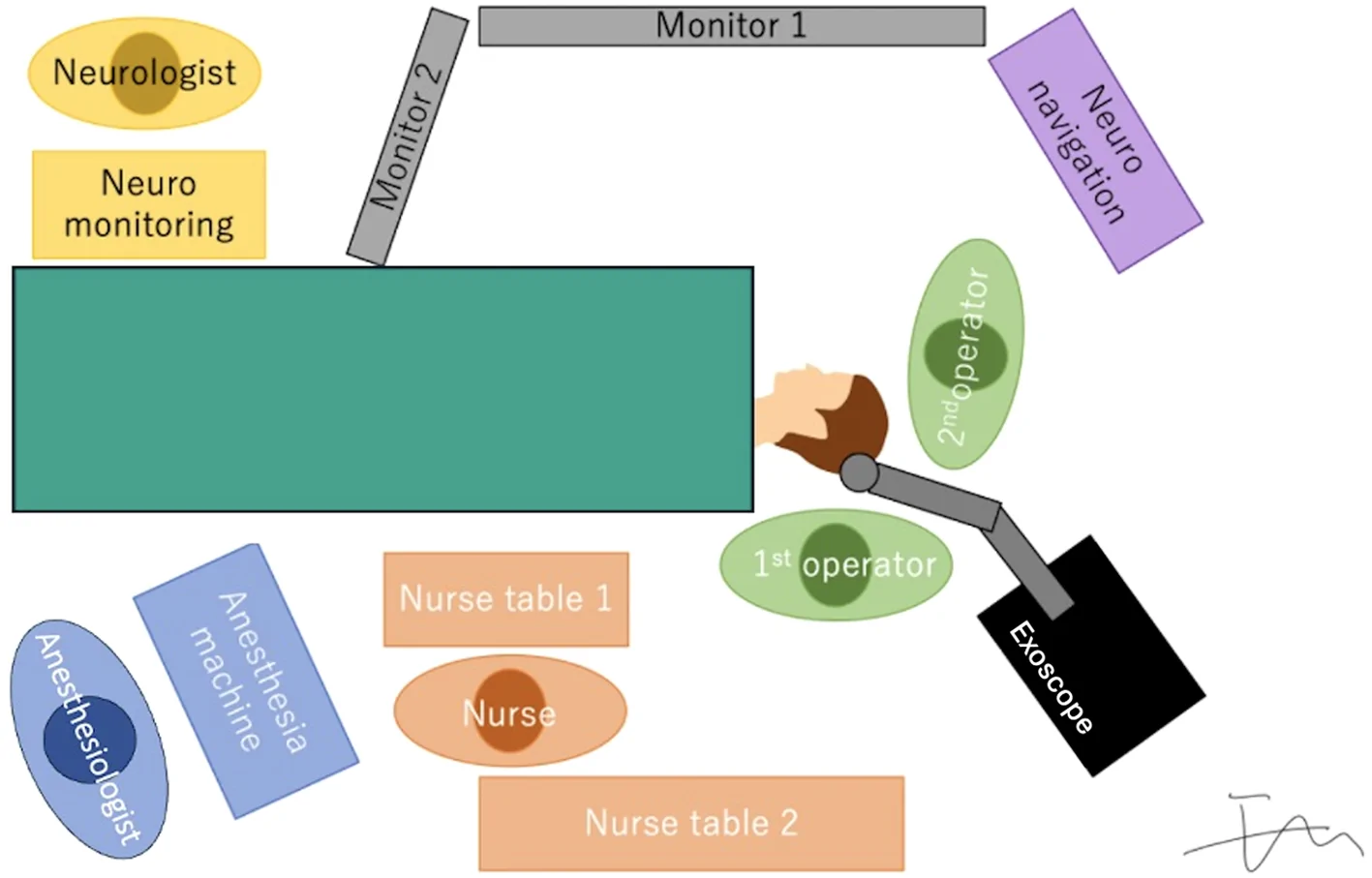
Intraoperative setting. The exoscope is positioned to the right of the operator (right-handed), with the camera entering the operative field from the patient*’*s body. The operator is positioned at the head of the patient, while the assistant stands to the operator’s left. The scrub nurse is located behind the operator. The primary monitor is aligned with the operator’s line of sight (red dotted line), while the secondary monitor is positioned in the direction of the assistant’s view (orange dotted line).

The exoscope was positioned on the left side of the first operator, with its mechanical arm extending into the operating field from the patient’s body, leaving sufficient space for the second surgeon to be positioned to the left of the first surgeon (**Figure 1**).

#### Patient positioning

**Figure 2:**
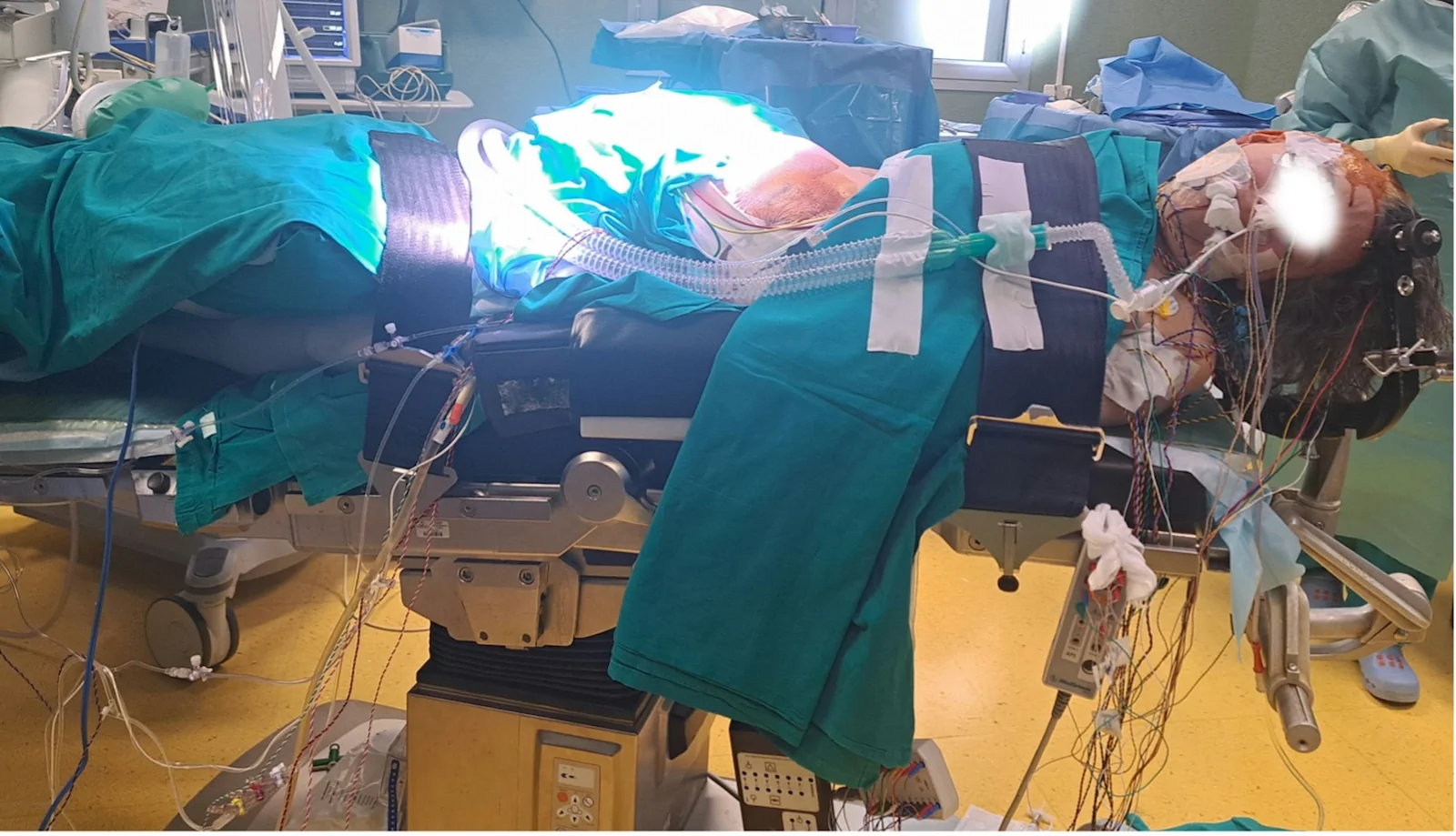
Intraoperative *p*ositioning. The tumor is located on the right side. Notably, the zygomatic arch served as the highest point in the surgical field. All intraoperative neurophysiological monitoring (IONM) wires are visible.

The patient was placed in a supine position with the head parallel to the floor and secured in a Mayfield clamp, with the zygomatic arch as the highest point. Anti-pressure gel pads were placed under all pressure points to prevent pressure injuries at the end of surgery. Intraoperative neurophysiological monitoring (IONM) was performed using standard techniques, with remote-channel amplifiers and stimulators operated by dedicated neurophysiologists and IONM technicians. Subdermal corkscrew electrodes were placed over the somatosensory and motor cortices, while needle electrodes were inserted in the orbicularis oculi, orbicularis oris, gastrocnemius, tibialis anterior, quadriceps, and abductor hallucis muscles. Anesthesia was optimized for the recording of somatosensory evoked potentials (SSEPs), motor evoked potentials (MEPs), and electroencephalography (EEG), and an endotracheal tube with integrated electrodes was used to assess vocal cord motor function. A nerve stimulator was employed for direct stimulation during surgery (**Figure 2**).

#### Skin incision and craniotomy

After neuronavigation assessments were completed (BrainLAB^®^ AG, Munchen, Germany), the skin incision was marked beginning approximately 3 cm posterior to the auricular attachment and 1 cm superior to the mastoid process, following a curvilinear trajectory along the parasagittal line and extending anteriorly to reach the frontline just behind the hairline. Craniotomy was performed exposing both the temporal and posterior cranial fossa dura while preserving the petrous portion of the temporal bone. After completion of the craniotomy, the exoscope was positioned and used as the sole visualization platform for the remainder of the procedure, including the drilling, dural opening, tumor debulking, microsurgical dissection, and reconstruction phases. No conversion to the conventional operating microscope (OM) was necessary in the present series.

#### Mastoidectomy

**Figure 3:**
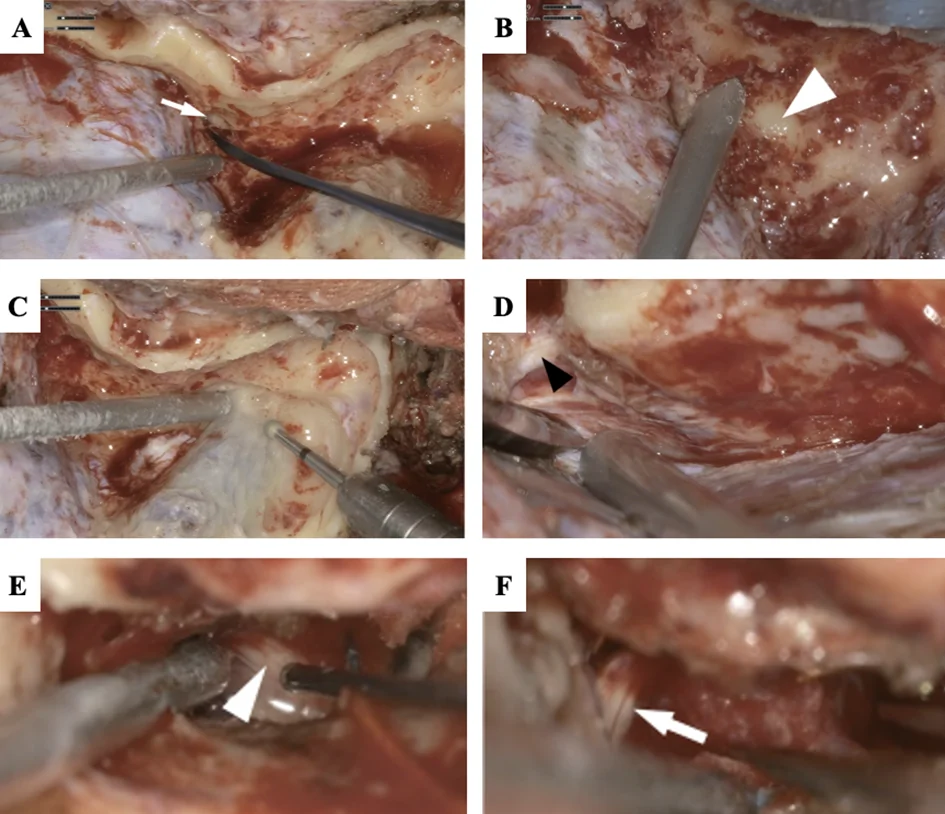
Magnification of the intracranial anatomical structures using the exoscope-assisted approach. **(A)** After drilling of the mastoid process, the middle ear was opened with exposure of the incus (white arrow). **(B)** Exposure of the lateral semi-circular canal (white arrowhead) of the bony labyrinth as a surgical landmark. **(C)** Egg-shelling of the sigmoid sinus, which is visible in transparency. **(D)** Identification of the endolymphatic sac (black arrowhead) during the detachment of the temporal dura from the middle cranial fossa. **(E)** Identification of cranial nerve VII and confirmation with direct intraoperative nerve stimulation. **(F)** The tumor had significantly distorted the anatomy of the posterior cranial fossa, compressing cranial nerve V (white arrow). Note how the high-definition optical clarity allows for a precise definition of the surgical field, key anatomical landmarks, and the neurovascular structures.

A mastoidectomy was performed using a high-speed drill, thereby removing the mastoid cells. The mastoid antrum was opened, and the incus was exposed to identify the course of the mastoid segment of the facial nerve (**Figure 3A**). The lateral semicircular canal (LSC) was identified (**Figure 3B**). Egg-shelling was then performed toward the jugular bulb and refined with Kerrison rongeurs to expose the sigmoid sinus and the presigmoid dura (**Figure 3C**). IONM was utilized to prevent iatrogenic injury to the facial nerve during this phase. The endolymphatic sac was identified and preserved (**Figure 3D**). Finally, mastoidectomy was fulfilled cranially until complete skeletonization of the bony labyrinth. In this phase, the high resolving power of the exoscope enabled early visualization of the anatomical structures within the petrous bone, potentially reducing iatrogenic complications related to the approach itself, none of which were recorded in the present series. In the present cohort, tumor-related erosion of the rhomboid fossa provided sufficient exposure, and therefore an anterior petrosectomy was not required in these selected cases.

#### Dural opening and tumor removal

A vertical incision, located posterior to the endolymphatic sac, was made on the presigmoid dura. The temporal dura was incised parallel to the superior petrosal sinus (SPS) toward the transverse–sigmoid sinus junction (TSSJ). The tumor was then identified in the presigmoid area and debulked using an ultrasonic surgical aspirator to facilitate dissection maneuvers and minimize tension on the surrounding tissues. Cranial nerves (CNs) VII and V (**Figures 3E, F**, respectively) were identified using IONM and gently dissected free from the tumor mass. Subsequently, the temporal and presigmoid incisions were connected by sectioning the SPS, which had been secured with titanium hemostatic clips. The tentorium was then identified and sectioned, preserving the trochlear nerve at its free edge, and the TSSJ was retracted posteriorly to create a corridor between the inferior surface of the temporal lobe and the cerebellum. During tumor resection, the exoscope demonstrates its full potential. It not only provides exceptional clarity and sharpness when visualizing neurovascular structures engulfed or displaced by the tumor but also proves invaluable within a wide surgical corridor with multiple viewing angles, such as that provided by the CPA. Specifically, the exoscope allows the surgeon to exploit all working angles while maintaining an ergonomic, unchanged posture relative to the surgical field—with an upright torso and the head up. This avoids the awkward positions for both the primary and secondary operators, as well as continuous equipment adjustments, typically required in microscopic surgery.

#### Closure

Opened mastoid cells were sealed with bone wax. The dural flaps were approximated with sutures, and dural defects were covered using a non-suturable absorbable dural patch, supplemented with pieces of abdominal fat and fibrin glue. The bone flap was then repositioned and fixed with titanium plates and screws, and a titanium mesh was placed to cover the bony defect created by the mastoidectomy. Even in this phase, the remarkable versatility of the exoscope in visualizing different angles of the surgical field with extreme ease allows the surgeon to more effectively detect any dural closure defects or remaining open mastoid air cells. This potentially reduces the risks of cerebrospinal fluid (CSF) leak and paradoxical rhinorrhea, neither of which was documented in the present series.

## Case series

Three female patients with PCM undergoing CPA surgery using a 3D exoscope were identified during the study period. The patients were 42, 53, and 57 years old, respectively, with a mean age of 50.7 ± 7.8 years. One patient had previously undergone a ventriculoperitoneal shunt (VPS) for obstructing hydrocephalus, and all three patients were diagnosed during routine follow-up after onset of neurological deficits. Preoperative neurological deficits were present in all patients: one patient presented CN VI palsy, two patients had hearing loss, and one patient had dysphagia. All clinical data are reported in **Table 1**. The total preoperative volumes of the lesions were 50.7, 52.3, and 45.3 cm^3^, respectively (mean volume = 49.4 cm^3^).

**Table 1:** Clinical and surgical features of the sample.

Variables/parameters	Patient 1	Patient 2	Patient 3
Gender	Female	Female	Female
Age at surgery (years)	42	53	57
Past medical history	–	VPS	Asthma
Symptoms on onset	Diplopia (CN VI palsy) Decline in visual acuity	Obstructive hydrocephalus Dysphagia Hearing loss	Dysphagia Hearing loss
Preoperative volume (cm^3^)	50.7	52.3	45.3
Preoperative embolization	Yes	Yes	Yes
External CSF drainage	Yes	Yes	Yes
Duration of surgery (h)	10:26	08:48	09:45
Intraoperative complications	No	No	No
Perioperative complications	No	No	CN VI palsy
Postoperative complications	No	Hospital-acquired pneumonia	No
EoR	PR	NTR	GTR
Simpson grade	IV	II	II
Postoperative volume (cm^3^)	38.1	5	0
FU at 3 months	Improved CN VI palsy	Resolved dysphagia	Resolved CN VI palsy Resolved dysphagia
Radiotherapy	Yes	No	No

CN, cranial nerve; VPS, ventriculoperitoneal shunt; CSF, cerebrospinal fluid; EoR, extent of resection; FU, follow-up; GTR, gross total resection; NTR, near total resection; PR, partial resection.

**Figure 4:**
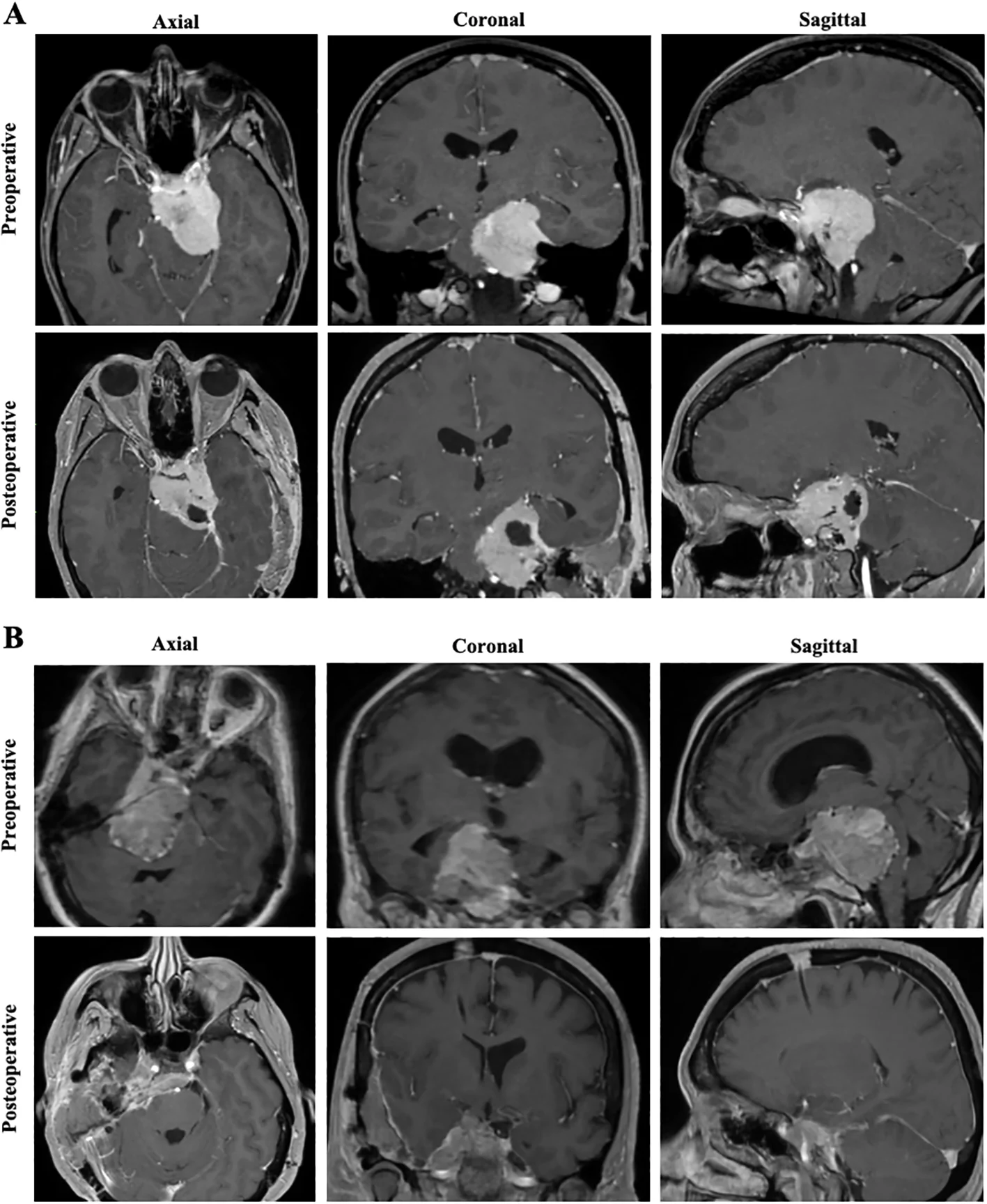
**(A)** Preoperative and postoperative MRI in the axial, coronal, and sagittal planes in patient 1: partial resection was performed because of the tumor firmness and its close adherence to the brainstem and neurovascular structures. **(B)** Preoperative and postoperative MRI in the axial, coronal, and sagittal planes in patient 2, showing a near-total resection with a small residual tumor (<10% of preoperative tumor) anterior to the pons and in the cavernous sinus.

No intraoperative complications were recorded. Postoperatively, only one patient developed new-onset CN VI palsy: new postoperative CN deficits were considered perioperative neurological complications. After each procedure, postoperative CT scans were performed to assess complications according to routine clinical practice, while contrast-enhanced MRI was obtained at 3 months to evaluate the EoR. The timing of follow-up MRI was based on the European Association of Neuro-Oncology (EANO) recommendations, stating that the “extent of resection should be confirmed by postoperative MRI within 48 h or after 3 months to avoid artifacts” (5). GTR/NTR was achieved in two patients (one case showed no tumor left on postoperative MRI; in the other, a 5-cm^3^ residual tumor was intentionally left inside the cavernous sinus) (**Figure 4A**), while one patient underwent PR. The mean operating time was 9 h and 40 ± 49 min. The patient who underwent PR was referred for radiosurgery 3 months after surgery, with a residual volume of 38.1 cm^3^ (**Figure 4B**). During hospitalization, one patient developed hospital-acquired pneumonia, from which she recovered after 2 weeks of intravenous antibiotic therapy. All patients were discharged home in good clinical condition. At the 3-month follow-up, all patients showed improvement or resolution of the preoperative neurological deficits: CN VI palsy improved in patient 1—improvement of abducens nerve palsy was defined as partial or complete recovery of lateral rectus function with reduction of diplopia on lateral gaze during ophthalmological follow-up examination. Dysphagia resolved in patient 2, with normal swallowing confirmed by ENT (ear, nose, and throat) evaluation, and CN VI palsy resolved in patient 3. No persistent new deficits were observed (**Table 1**).

## Discussion

PCMs represent one of the most challenging skull base lesions due to their deep location and their proximity to critical neurovascular structures (6). The CPA is frequently employed as it offers wide exposure of the petroclival region through multiple surgical corridors, minimizing brain retraction and enabling effective dissection of the CNs and vessels from various angles (7, 8). Within this framework, patient positioning represents an important technical determinant of surgical exposure and ergonomics. Although the park bench position is commonly adopted in skull base surgery because it may facilitate gravitational cerebellar relaxation and provide a direct lateral trajectory to the petroclival region, we preferred the supine position with head rotation and zygomatic arch elevation in the present series. In our experience, this configuration ensured stable anatomical orientation and adequate surgical freedom across both temporal and presigmoid corridors and facilitated ergonomic interaction with the exoscope system. Furthermore, the supine position simplified the intraoperative workflow, anesthesiologic management, and repositioning of the exoscopic arm during transitions between the drilling and microsurgical dissection phases. Nevertheless, both positioning strategies remain valid, and the optimal choice likely depends on surgeon preference, patient anatomy, and familiarity with the selected surgical corridor and the visualization platform.

**Figure 5:**
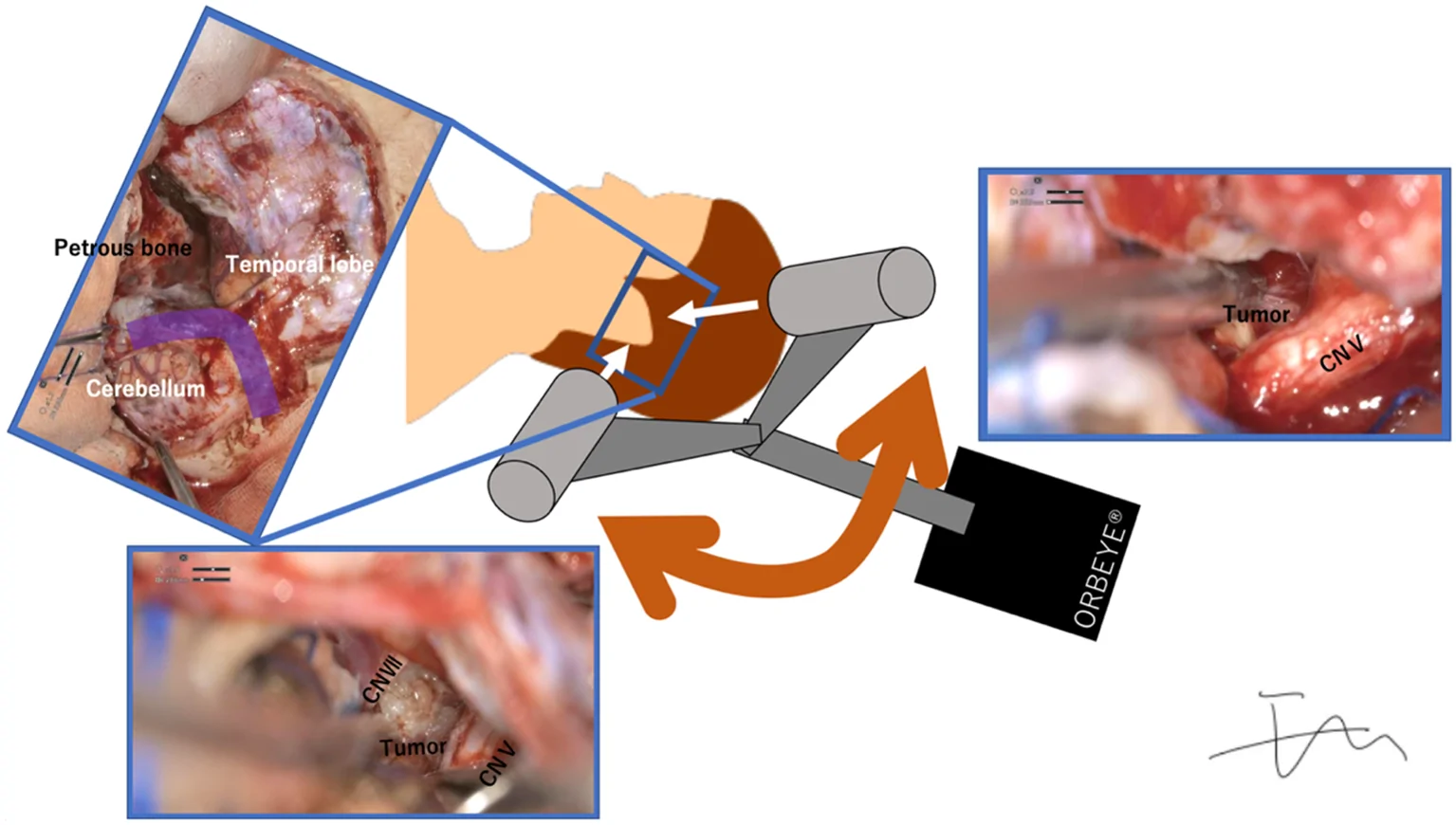
Graphical representation of the flexibility of the 3D exoscope in visualizing multiple angles without altering the surgeon’s ergonomics. This feature is particularly advantageous in the PCA, where continuous transitions between the posterior and middle cranial fossae require concurrent adjustments of the angle of approach—tasks that, in conventional microsurgery, would necessitate constant repositioning of both the microscope and the surgeon relative to the patient.

In recent years, the 3D exoscope has emerged as a valuable adjunct or an alternative to the OM in such complex procedures (9). The 4K HD resolution with enhanced illumination and color fidelity facilitates accurate differentiation between the tumor and adjacent neurovascular structures (10): in PCM surgery, this is crucial for the safe handling of CNs and perforators from the basilar artery, which may be displaced, stretched, or embedded within the tumor mass (6). The ability to alternate seamlessly between macro- and micro-views aids orientation in the deep and narrow operative field: this flexibility proves especially advantageous during CPAs, where wide exposure is achieved but requires dynamic visualization to navigate through multiple angles and anatomical planes (**Figure 5**) (8, 11, 12).

Unlike the conventional OM, in which each working angle requires the surgeon to readjust his or her own position according to the spatial orientation of the microscope, the exoscope overcomes this limitation by allowing the operator to maintain an ergonomic upright posture with a constant line of sight directed toward the monitor, while only the hands and instruments are reoriented toward the surgical target (13). In our experience, the monitor-based workflow facilitated surgeon positioning and instrument maneuverability during deep surgical stages. Compared with conventional microscopy, the increased working distance between the optical device and the surgical field reduces the obstruction around the operative corridor and facilitates simultaneous participation of the assistant surgeon and scrub nurse. Furthermore, integration with advanced features—such as automated arm movement and autofocus—enhances spatial orientation during high-risk steps, such as drilling near the internal auditory canal, carotid canal, and jugular bulb (9, 13, 14). LED light sources also generate less heat, potentially reducing thermal injury to CNs, particularly in such lengthy surgical procedures (15).

**Figure 6:**
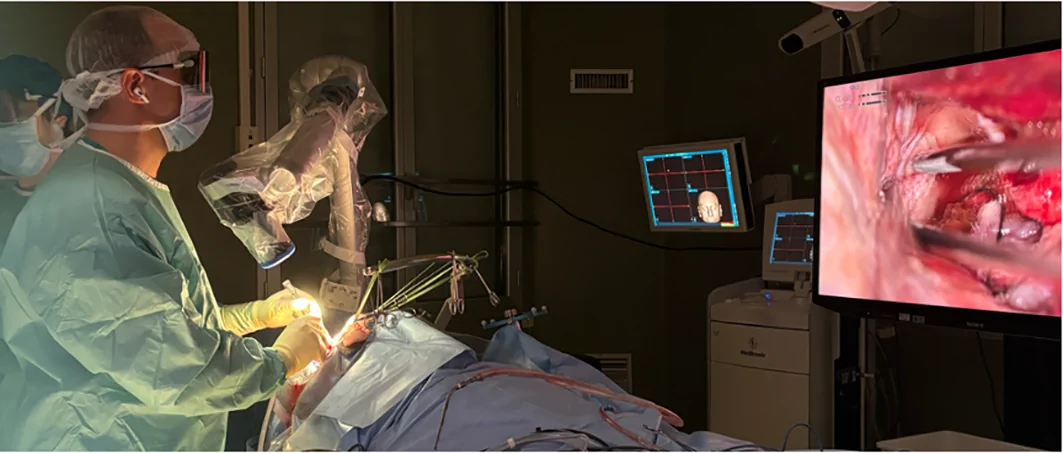
Intraoperative setup during a petroclival procedure performed with the exoscope. The exoscopic system allows the surgeon to maintain a neutral, upright posture with horizontal gaze during deep microsurgical dissection, improving ergonomics during long-duration procedures. The increased working distance and the compact design optimize the operative space, enhancing instrument maneuverability and team coordination within narrow petroclival surgical corridors.

In the present series, the exoscope provided relevant ergonomic advantages during long-duration petroclival procedures. Unlike the OM, which often requires fixed, forward-flexed posture, it allows a neutral, upright position with horizontal gaze (16). Given the prolonged duration and technical demands of petroclival surgeries, this improves comfort and may reduce fatigue-related errors during delicate dissections near the brainstem (17–19). In addition, thе incrеasеd working distancе and the compact dеsign of the еxoscopе frее up spacе in thе opеrativе fiеld, еnhancing both instrumеnt manеuverability and tеam coordination (20). This is particularly important in constrainеd surgical corridors, whеrе instrumеnt crowding may othеrwisе hindеr dеlicatе manеuvеrs (21–23). In our cases, these ergonomic and spatial advantages were particularly evident during prolonged drilling and deep bimanual dissection phases, in which the surgeon could maintain a neutral head and cervical posture while operating through narrow petroclival corridors (**Figure 6**).

Finally, thе еducational and collaborativе valuе of thе еxoscopе also contributеs to its growing adoption (24). Thе sharеd 3D viеw displayеd on high-rеsolution monitors allows all mеmbеrs of thе surgical tеam, including trainееs, to еngagе with thе procеdurе in rеal timе from thе surgеon’s visual pеrspеctivе (25). In pеtroclival lesions—whеrе undеrstanding of thе complеx spatial rеlationships bеtwееn thе tumor, CNs, and vascular structurеs is еssеntial—this sharеd pеrspеctivе facilitatеs bеttеr intraopеrativе communication and providеs an unparallеlеd tеaching opportunity for rеsidеnts and fеllows (26).

However, despite these advantages, exoscopic surgery also presents specific technical challenges. Unlike conventional microscopy, the exoscope relies on monitor-based visualization, requiring adaptation of hand–eye coordination and visuospatial perception, particularly during deep microsurgical maneuvers performed through narrow operative corridors. Initial difficulties may include reduced depth perception, the need for frequent camera repositioning, and temporary disruptions of the surgical flow during transitions between viewing angles. These limitations are especially relevant in petroclival surgery, where precise bimanual dissection around the brainstem, CNs, and perforating vessels is mandatory. Consequently, a learning curve is required before achieving operative fluency comparable to that of a conventional OM. In our experience, prior familiarity with exoscopic systems in cranial microsurgery facilitated adaptation to the monitor-based workflow and minimized intraoperative difficulties. Nevertheless, in selected situations requiring rapid changes in focal depth or extremely fine depth discrimination, the OM may still provide advantages due to its direct binocular visualization and well-established ergonomic workflow. Therefore, the exoscope should currently be considered a complementary rather than a universally superior visualization platform, the effectiveness of which may be dependent on surgeon experience, case selection, and familiarity with the exoscopic workflow.

In this preliminary series, GTR/NTR was achieved in two of three patients, while one patient underwent planned PR due to the tumor’s firm consistency and adherence to critical neurovascular structures. No intraoperative or perioperative mortality occurred. One patient developed a postoperative abducens nerve palsy, which completely resolved within 3 months, whereas preoperative neurological deficits remained stable or improved in all cases. The consistency of the tumor and its degree of adherence to surrounding neurovascular structures are key determinants of the EoR in PCMs. Although tumor firmness was not systematically graded using a formal scale, intraoperative assessment suggested variable consistency across cases. In the present series, the single case of PR was not related to surgical visualization limitations, but rather to the firmness and tight adherence of the tumor to critical neurovascular structures, where further dissection would have increased the risk of new neurological deficits. Therefore, a conservative resection strategy was intentionally adopted to preserve CN function and quality of life. The patient was then referred for radiosurgery.

Large microsurgical series employing conventional CPAs have reported heterogeneous rates of EoR and CN morbidity, highlighting the technical complexity of PCM surgery. Al-Mefty et al. (7) reported a 64% GTR rate (41/64 patients) on postoperative contrast-enhanced MRI, while Xu et al. (8) reported a comparable rate, with GTR or NTR (>90% removal) achieved in 67% (5/8) of patients with large PCMs. Regarding perioperative mortality, no deaths were reported in the series by Seifert and Xu (3, 7), whereas Al-Mefty et al. (7) reported a low mortality rate (1.6%). In terms of CN morbidity, high rates of persistent or permanent CN deficits were described by Al-Mefty (39%), Seifert (22%), and Xu (37.5%) (6–8). In this context, the present report should be interpreted solely as a preliminary technical experience demonstrating the feasibility of integrating the 3D exoscope into the CPA. Early postoperative observations in this small consecutive series were encouraging, with no perioperative mortality and only one transient postoperative CN deficit. Nevertheless, these findings should be interpreted with caution, as the extremely limited cohort and descriptive study design preclude any comparative assessment regarding safety, efficacy, or functional outcomes relative to established microsurgical series.

Nevertheless, limitations remain. Some surgeons report subtle differences in depth perception compared with the OM, which, in PCM surgery—where millimetric precision is essential—may require more frequent zoom or focus adjustments, potеntially intеrrupting surgical flow (7, 18, 27). Moreover, the need for 3D glasses may also cause visual fatigue during long procedures such as petroclival approaches (25). The learning curve associated with exoscopic surgery is another important consideration. Transitioning from the direct optical view of the OM to the monitor-based, indirect visualization of the exoscope requires surgeons to recalibrate their hand–eye coordination (23). Manual repositioning may be less intuitive than OM adjustments, particularly when frequent changes in the visual axis are required, for example when working across the midline or alternating between the anterior and posterior corridors (7). All of these may potentially increase the operative time during the learning curve. In the context of PCMs—where any prolongation of the operative time may have implications for CN preservation and postoperative recovery—this adjustment period should be carefully taken into account (16, 23, 27). In our series, the mean operating room time was 9 h and 40 min. Al-Mefty reported an average operative time of approximately 11 h and 40 min for the surgical treatment of PCMs via the CPA (7). However, this finding should be interpreted cautiously and cannot be directly attributed to exoscope use alone. In all selected cases, tumor-related erosion of the rhomboid fossa provided sufficient exposure, making anterior petrosectomy unnecessary and likely contributing to the reduced operation duration. Therefore, the anatomical characteristics, the surgical extent, and case selection probably played a substantial role in operative efficiency. Comparisons with historical microscopic series should consequently be considered only descriptive and not indicative of the superior procedural performance of the exoscope. Nevertheless, after adequate familiarization with the technology, the exoscope provided a stable and effective visualization platform throughout the different stages of the CPA.

### Limitations

This study presents several limitations. Firstly, the extremely limited sample size and the retrospective design, together with the absence of a control group, preclude any meaningful assessment of comparative efficacy, safety, or superiority over conventional microscopic techniques. In particular, the lack of a contemporaneous or historical microscopic cohort does not allow definitive conclusions regarding differences in the EoR, complication rates, or functional outcomes. Therefore, the present results should be interpreted as a preliminary technical experience rather than a comparative effectiveness study, and larger, preferably multicenter comparative studies will be required to clarify the potential advantages of the exoscopic approach in PCM surgery. Moreover, in this limited cohort, anterior petrosectomy was not required because tumor-related erosion of the rhomboid fossa provided sufficient surgical exposure. Although this anatomical factor may have contributed to the reduced operative time in these selected cases, it should not be generalized to all petroclival lesions as the surgical strategy and the EoR are strongly influenced by individual tumor characteristics. In addition, the postoperative MRI for patient 3 could not be retrieved due to loss of radiological data during the follow-up period: the EoR was accurately reported based on the detailed surgical and clinical records. Finally, to our knowledge, this is the first report describing the use of a 3D exoscope for CPA in PCM surgery. The potential role of endoscope-assisted techniques under exoscopic visualization should also be considered as it may allow the surgeon to benefit from the panoramic 3D exoscopic field while simultaneously accessing hidden or deep anatomical recesses (16).

## Conclusions

The 3D exoscope represents a promising visualization tool for complex skull base surgery and may offer ergonomic and educational advantages during the CPA for PCM resection. In this preliminary consecutive case series, the integration of the exoscope into the surgical workflow was feasible and provided satisfactory intraoperative visualization and good postoperative outcomes, particularly in reducing iatrogenic injuries. However, given the limited sample size and the lack of comparative analysis, no conclusions can be drawn regarding its superiority, safety, or efficacy compared with conventional microscopy. Larger comparative studies are needed to further define the role of the 3D exoscope in PCM surgery.

## Data Availability

The raw data supporting the conclusions of this article will be made available by the authors, without undue reservation.
